# Moderating Effects on the Link between Violent Pornography and Sexual Aggression

**DOI:** 10.1007/s10508-025-03199-y

**Published:** 2025-07-22

**Authors:** Melissa S. de Roos, Emma Ferrando

**Affiliations:** Department of Psychology, Education & Child Studies, Erasmus School of Social and Behavioural Sciences, Erasmus University Rotterdam, Burgemeester Oudlaan 50, 3062 PA Rotterdam, The Netherlands

**Keywords:** Violent pornography, Sexual violence, Rape myth acceptance, Sexual script theory, Pornography

## Abstract

Sexual violence remains a widespread problem among university students, with negative consequences ranging from mental and physical health problems to academic effects and interpersonal issues. Sexual scripts form the blueprint for sexual interactions. Such scripts are influenced by personal experience, as well as exposure to external sources. One such source is violent pornography, with its effect on sexual violence perpetration established in various studies. The aim of this study was to examine the link between violent pornography consumption and sexual violence perpetration. Further, we examined the moderating effects of perceived pornography realism and peer rape myth acceptance (RMA). University students (*N* = 686, 63.4% female) from The Netherlands participated in this online survey study. Male participants held more positive views toward pornography, viewed more frequently and more deviant content, and their perceived peer RMA was higher than that of women. Results indicated that, particularly for male students, viewing violent pornography increased the risk of sexual violence perpetration. This effect was further exacerbated if pornography was perceived as realistic and peer RMA was high. No such association between violent pornography and sexual violence perpetration was found for women, but this link was observed if peer RMA was high. Implications for prevention focusing on porn literacy initiatives and peer groups are discussed.

## Introduction

A substantial proportion of children and adolescents start viewing pornography at a young age (e.g., Bernstein et al., 2023; Children’s Commissioner, 2023; Giordano et al., 2022). Notably, pornography often includes violence (Bridges et al., 2010). Fritz et al. (2020) analyzed more than 4000 scenes from two mainstream pornographic websites (Pornhub and Xvideos). They found that physical aggression was present in 35–45% of scenes. The most depicted violent acts were spanking, slapping, gagging, and hair-pulling (Fritz et al., 2020). These findings suggest that a considerable percentage of young individuals are likely to be exposed to violent pornography.

Drawing upon sexual script theory, which poses that a combination of personal experience and outside influence shapes our expectations about sexual situations (Simon & Gagnon, 2003), this (early) exposure to pornography could impact how individuals perceive sexual interactions. Indeed, the 3AM theory (Wright, 2011) details the process by which media consumption becomes incorporated in sexual scripts, and violent pornography consumption was added as a significant contributing factor to the confluence model, which seeks to explain male-perpetrated sexual aggression (Malamuth et al., 2021). University students are among the top consumers of online pornography (Price et al., 2016) and face elevated risks of sexual assault. Around 20% of women and 10% of men report experiencing unwanted sexual contact during their time at university (Muehlenhard et al., 2017). These incidents can lead to serious long-term effects, including depression, PTSD (Lindquist et al., 2013), anxiety (Carey et al., 2018), and heightened vulnerability to future victimization (Fisher et al., 2000). The focus of this study is on examining how cultural (pornography) and interpersonal scripts (peer interaction) influence sexual scripts and contribute to a potential risk of sexual violence perpetration.

### Violent Pornography

As a cultural factor, mainstream media such as pornography, plays a critical role in the development of sexual scripts (Bridges et al., 2016). Pornography consumption has been associated with the adoption of unrealistic attitudes about sex and less progressive gender role attitudes (Horvath et al., 2013; Koletić, 2017; Peter & Valkenburg, 2016). Notably, the content of pornographic often aligns with themes that are presumed to cater to the preferences of a predominantly male audience (Bareket & Shnabel, 2020; Klaassen & Peter, 2015,), often featuring themes such as domination and sexual objectification (Sun et al., 2016), where women are portrayed as “tools” for male gratification (Bridges et al., 2010). Of even greater concern, pornography often portrays violence, with women as the objects of degradation (Sun et al., 2016) and as experiencing pleasure from aggression (Bridges et al., 2010). Given that young individuals may view pornography to learn skills, sexual roles, and how to behave during sexual encounters (Horvath et al., 2013; Rothman et al., 2021), exposure to violent sexual content may contribute to the legitimization of coercive and violent sexual behaviors (Matković et al., 2018) and the perpetuation of harmful gender stereotypes, offering a pivotal perspective to understanding sexual aggression perpetration (O’Connor et al., 2021; Tomaszewska & Krahé, 2018).

Indeed, consumption of violent pornography has been linked to sexual aggression perpetration (Wright et al., 2016). Violent pornography specifically was associated with and predicted later sexual aggression perpetration in both men and women (Rostad et al., 2019; Ybarra et al., 2011), with some studies reporting that consuming sexually violent pornography was associated with sexual aggression, whereas consuming non-violent pornography was not (Ferguson & Hartley, 2022; Ybarra et al., 2011). The link between violent pornography consumption and sexual aggression seems stronger for men. College men’s use of sexually violent pornography was associated with sexual aggression perpetration and their self-reported likelihood to commit rape (Rodenhizer & Edwards, 2019). Since sexually graphic media most frequently depicts male-perpetrated violence, it may be easier for (young) men to relate to the perpetrator in such scenarios (Cohen, 2001), which could make such information more readily integrated into sexual scripts.

Although the literature supports a link between violent pornography consumption and the likelihood of sexual aggression perpetration, mere exposure to media depicting violence does not cause aggressive behavior (Anderson et al., 2015). Instead, individuals with preexisting violent sexual scripts may be more likely to seek out violent sexual content (Haridakis, 2002; Zurbriggen & Morgan, 2006) and be influenced by it **(**Horvath et al., 2013). Nonetheless, exposure to violent pornography may further desensitize viewers and legitimize specific sexual elements (Wright, 2011), including the use of violence to seek sexual pleasure (Rodenhizer & Edwards, 2019). Thus, viewing violent pornography may validate and reinforce an individual’s existing aggressive sexual scripts (Rodenhizer & Edwards, 2019). Further, various moderators have been identified that affect any link between violent pornography consumption and sexual violence perpetration.

### Perceived Pornography Realism

Perceived realism of consumed pornography is a moderating factor in the link between violent pornography consumption and sexual violence perpetration (Krahé et al., 2021; Tomaszewska & Krahé, 2018). The degree to which someone regards pornography as reflective of real life denotes an individual difference, whereby a greater perceived realism likely strengthens any link between violent pornography consumption and sexual violence perpetration (Gunnoo & Powell, 2023). Although Löfgren-Mårtenson and Månsson (2010) found that adolescents could differentiate between pornographic content and real-world sexual encounters, pornography appears to be, to some degree, a shared learning manual, with its content being integrated into individuals’ sexual scripts.

To an extent, perceived realism ties in with frequency of consumption. In general, frequent media exposure can influence reality perception as the viewer will start to believe that the social norms portrayed are accurate (Gerbner et al., 1994). The same is true for pornography. In fact, frequency of pornography use has been associated with an increase in the perceived realism of its content (Peter & Valkenburg, 2006), and higher pornography use was linked to increased interest and engagement in pornographic sexual behavior (Sun et al., 2016), such as spanking, being choked, or degrading sexual acts (Bridges et al., 2016). Through increased viewing, pornography may begin to seem more realistic because of its apparent congruence with developing scripts that normalize (violent) behaviors and portray these as rewarding to the individual actors in such material. This increase in perceived realism in turn heightens the chance of the person re-enacting the behaviors depicted (Peter & Valkenburg, 2006, 2010). In a similar vein, a longitudinal study examining the development of pornography consumption found that perceived realism impacted the link between pornography consumption and impersonal sexual attitudes (Baams et al., 2015). Impersonal sexual attitudes in turn constitutes one of the main pillars of the confluence model explaining sexual aggression in men (Malamuth et al., 2021).

The more content is perceived to be realistic, the more likely it is internalized as normative (Bandura, 1976; Peter & Valkenburg, 2010). Hence, individuals who frequently consume violent pornography may internalize distorted beliefs about sexual interactions, potentially resulting in the legitimization of violence and degradation during sexual encounters. For example, a study by Zhang et al. (2024) found that perceived pornography realism moderated the link between pornography use and hostile sexism. Indeed, research supports perceived realism as a critical factor in predicting sexual aggression perpetration through its influence on sexual scripts (Baams et al., 2015; Krahé et al., 2021). Although Krahé et al. (2021) found that men perceive pornography as more realistic than women, the link between perceived pornography realism and sexual aggression was found in both genders, whereas other studies have failed to find a difference in perceived realism ratings (Baams et al., 2015; Wright & Štulhofer, 2019; Wright et al., 2021).

### Peer Rape Myth Acceptance

Beyond mass media, and how individuals perceive such media, sexual scripts are also affected by social networks (Gagnon, 1990). In the literature, peer influence is a recurring motif in shaping sexual norms and attitudes. College students who reported having a friend circle accepting of casual sex and hookups were more likely to have casual sex and more sexual partners (Trinh et al., 2014) or to participate in hookups (Holman & Sillars, 2012). Similarly, having a friend circle promoting abstinence was linked to less dating and sexual experience in a college sample (Trinh et al., 2014). Perception of friends’ approval of sexual violence (Collibee et al., 2021; Foshee et al., 2013) and own engagement in aggressive sexual behavior (Capaldi et al., 2012) also predicted the perpetration of dating violence among adolescents. Thus, peer messages endorsing sexual aggression can potentially increase the risk of sexual aggression perpetration.

Rape myths in particular have been associated with a rape culture that contributes to the acceptance of sexual violence (Trottier et al., 2021). Rape myths are beliefs that blame the victim, absolve the perpetrator, and trivialize sexual assault incidents that do not conform to the “real rape” narrative—i.e., rape is physical and violent, and the assailant is usually a stranger (Horvath & Brown, 2009)—(Payne et al., 1999). Accordingly, rape myth acceptance (RMA) has been associated with victim-blaming attitudes, acceptance of interpersonal violence—including sexual aggression, coercion, and rape—and male dominance attitudes (Suarez & Gadalla, 2010).

When these harmful beliefs are condoned and integrated into sexual scripts, they may normalize sexually aggressive behavior. Men may use rape myths as criteria for identifying situations where sexual coercion is justifiable and tolerated (e.g., it is okay to rape women who have shown sexual interest; Fernández-Fuertes et al., 2020). Notably, men with high RMA appear to be less sensitive at detecting rejection cues (Farris et al., 2006), suggesting that they may hold a “real rape” script which, in turn, justifies their persistence. Moreover, evidence shows that higher RMA is associated with an increased risk of sexual aggression perpetration (O’Connor et al., 2021; Trottier et al., 2021).

As peer messages influence sexual behavior (Collibee et al., 2021; Foshee et al., 2013; Holman & Sillars, 2012; Trinh et al., 2014), it is plausible that having a social circle that endorses rape myths has an impact on an individual’s own RMA and their risk of sexual aggression perpetration. Accordingly, Bohner et al. (2006) found that in a sample of male college students, respondents’ perceptions of their peers’ RMA influenced their own beliefs, with greater peer acceptance predicting higher personal acceptance. Furthermore, this increased peer influence indirectly raised the respondents’ reported rape proclivity by shaping their own RMA (Bohner et al., 2006). Whereas individuals’ own RMA has been studied in conjunction with violent pornography consumption, no studies have looked at perceived peer RMA in this context.

### The Present Study

Although substantial research has studied the effects of violent pornography consumption on individual behavior, few studies have simultaneously assessed individual (perceived realism) and social (peer RMA) moderating factors. Such parallel moderation models are relatively rare, as most research tends to isolate one moderating factor without considering how multiple moderators independently influence the link between violent pornography consumption and sexual violence perpetration. Existing research has not extensively addressed the nuanced ways in which individual and social factors interact, or the extent to which such interactions differ between men and women. Further, less is known about the role of peer networks in shaping the link between pornography consumption in sexual aggression, and perceived pornography realism similarly is a comparatively understudied factor that specifically touches upon individuals’ beliefs about the pornography they consume. By adding this factor as a moderator, we hope to provide greater insight into how belief systems regarding pornography can influence the risk of sexual violence perpetration. Finally, studying these associations in a sample of university students offers a unique perspective on sexual scripts among young adults, given the proximity of peer networks and increased opportunities to engage in sexual behaviors (Berntson et al., 2014). Given these gaps in the literature, the purpose of this study is to investigate the link between violent pornography and sexual violence perpetration, and the moderating effects of peer RMA and perceived realism.

We hypothesized that (H1) men would view more pornography and have more positive attitudes toward it than women; (H2) men would perpetrate more sexual aggression than women; (H3) men would report higher peer RMA; (H4) for both men and women, viewing violent pornography would increase the risk of sexual aggression perpetration, (H5) particularly if peer RMA and (H6) perceived pornography realism are high.

## Methods

### Participants

A link was distributed via the university research participant platform at a Erasmus University Rotterdam, through flyers on campus, and on various social media networks (Reddit, X, Facebook, and LinkedIn). All participants over age eighteen were eligible to participate. The link was accessed by 856 participants. We deleted 127 participants who had missing data, a further 44 who finished the survey in less than five minutes and nine participants who were not currently students. The final sample consisted of 677 participants (64.1% female, 29.8% male, 1.6% non-binary). Participants ranged in age from 18 to 42 years (*M* = 22.05, *SD* = 3.38). Our sample was mostly heterosexual (70.9%), with 15.8% identifying as bisexual and 6.6% as gay or lesbian. Just over half of our sample indicated they were currently dating (9.7%) or in a relationship (43.6%).

### Measures

#### Illinois Rape Myth Acceptance Scale–Short Form (Payne et al., 1999)

This scale measures RMA using twenty items. Three of these are filler items, which were removed before analyses took place. We modified this scale, giving participants the following instruction: “Think about your three closest friends. Imagine you are having a private conversation with them. To what extent do you think they would agree with the following statements.” Sample items include “Women tend to exaggerate how much rape affects them” and “Men don’t usually intend to force sex on a woman, but sometimes they get too sexually carried away.” Participants answered on a 5-point Likert scale (1 = *Strongly Disagree* to 5 = *Strongly Agree*) and responses were summed, with higher scores indicating greater peer RMA. A modified version of the IRMA has been used in prior research to assess peer acceptance of rape myths, and it has shown good internal reliability (α = 0.82; Collibee et al., 2021). Internal consistency in this sample was excellent α = 0.91.

#### Pornography Consumption Questionnaire (Hald, 2006)

This scale first provides participants with a standardized definition of pornography: “any kind of material aiming at creating or enhancing sexual feelings or thoughts […] containing explicit exposure and/or descriptions of genitals and clear and explicit sexual acts […].” Following this, various questions are asked about frequency of use (age at first use, average use per week, use in the last six months, last use), and how acceptable and realistic participants deem pornography to be (1 = *Not at all,* to 5 = *Entirely*). Finally, participants report the types of pornography they view (vaginal penetration, anal penetration, oral sex, teens, group sex, fetish/BDSM, and violent sex/rape scenarios), with each of these items also scored on a 5-point Likert scale (1 = *Never used*, to 5 = *Always used*). The scale’s validity has been assessed as good (Hald et al., 2013).

#### Sexual Perpetration

Sexual perpetration was assessed using the Sexual Experiences Survey—Short Form Perpetrator (Koss et al., 2007). We modified the instructions to ask participants about experiences while they were a student. This questionnaire asks about seven behaviors (fondling/undressing, (attempted) oral sex, (attempted) vaginal penetration and (attempted) anal penetration). Participants then report how often (0, 1, 2, 3 + times) they engaged in the behavior, through coercion, while the victim was incapacitated, or through (threats of) violence/use of force. Participants were then placed into mutually exclusive categories to indicate their most severe behavior (non-perpetrator, sexual contact, attempted coercion, coercion, attempted rape, rape). Further, participants are asked whether they believe they have ever raped someone (Yes/No), which allows us to compare people who admit to explicitly mentioned “rape” with those who meet the behavioral criteria of rape based on their answers to the earlier items. Finally, participants are asked the number of times participants engaged in any of the specified behaviors, and the gender they perpetrated these acts to (men only/women only/both). The SES-SFP has demonstrated validity and reliability (Johnson et al., 2017).

### Procedure

When participants accessed the link, they were first presented with an informed consent form, which also listed contact information for various organizations relating to support in cases of sexual violence. Following this, participants filled out questionnaires in a randomized order. The study was part of a larger study which also included questionnaires about recognizing and responding to risky sexual situations. Upon completion, participants were presented with a debrief form, which again listed contact information for various supporting organizations.

### Data Analysis

Listwise deletion was used to handle missing data, as missing data on any individual item did not exceed 5% and the data were missing at random. To assess H1, H2 and H3, we calculated descriptive statistics or frequencies for all variables. For the perpetration, we followed both coding instructions provided by Koss et al. (2007), which first creates non-redundant scores by placing each participant in the most severe category of behavior they had perpetrated. If a participant answered affirmatively to having attempted coercion, but had also engaged in rape, they would be placed in the rape category. As such, these categories are mutually exclusive (each participant is categorized once, according to their most severe behavior), but due to missing data, they did not add up to 100%. We chose to use the mutually exclusive scoring method to create an index of severity of perpetration. However, upon inspection of the data, this scoring method placed nearly all participants either in the non-offending category or in the most severe offending category of rape. As a result, we decided to dichotomize the variable into yes or no, because there was very little variability to justify using the full spectrum. For illustration purposes, we also include the absolute prevalence of each category of behavior in our results.

We then calculated Pearson correlations for the associations between pornography variables and total rape myth acceptance, and point biserial correlations for the associations between pornography variables and sexual perpetration (dichotomized). For the pornography content questions, we used the full range (never to always) rather than the dichotomized variables. Finally, to assess H3, H4 and H5, we conducted a series of logistic parallel moderation analyses with perpetration (DV), violent pornography (IV), perceived realism (M1) and peer RMA (M2). We selected a logistic parallel moderation model because it allows assessment of how the relationship between violent pornography (IV) and sexual aggression perpetration (DV) is influenced by the moderators (perceived realism and peer RMA). Given our research questions, which seek to explore the interactive effects of consumption of sexual media on the one hand and peer beliefs and perceived realism on the other hand in predicting sexual aggression, a moderation model is most appropriate. This model allows investigation of whether the effect of violent pornography on sexual aggression varies across different levels of perceived realism and peer RMA. We conducted one moderation analysis for the full sample of men, one for the full sample of women, and one for the sample of women who watch pornography. In using these three samples, we can examine potential gender differences and explore the role of pornography consumption in a subgroup of women who engage with pornography. The findings from these analyses will directly address our hypotheses that violent pornography viewing increases the risk of sexual aggression, particularly if peer RMA and perceived realism are high.

## Results

### Descriptive Statistics

To assess the first hypothesis that men would view more pornography and have more positive attitudes toward it than women, and the third hypothesis that men would score higher on peer RMA, we calculated descriptive statistics of pornography variables and peer RMA. Results are displayed in Table 1. Only three men (1.5%) and 83 women (19.1%) indicated they had never viewed pornography. Only people who indicated they viewed pornography were included in the subsequent analyses that included pornography consumption, unless otherwise specified. Consistent with our expectations, men scored higher than women on peer RMA, positive attitudes toward pornography, and frequency of use. However, overall, peer RMA was low, and the difference is between Strongly Disagree (women) and Disagree (men). Frequencies of viewing various types of pornography are displayed in Table 2. Men watched significantly more of every type of pornography than women except for vaginal penetration.

**Table 1:** Independent samples *t* tests of the differences in peer rape myth acceptance and pornography variables between men and women

	Men	Women	*t*
	M (SD)	M(SD)	
Peer RMA	36.81 (13.49)	24.13 (10.13)	11.86***
Acceptable	3.10 (.64)	2.90 (.67)	3.36***
Realistic	2.53 (1.24)	1.80 (.98)	7.13***
Past six months	4.05 (1.25)	2.76 (1.15)	12.16***
Hours a week	2.74 (.92)	2.10 (.73)	8.34***

Scores on Acceptable ranged from 1–4 with higher scores indicating greater acceptability, Realistic ranged from 1–5 with higher scores indicating greater realism, and the remaining scores ranged from 1–6 with higher scores indicating greater frequency

***p < .001

**Table 2:** χ^2^ tests of differences in viewing specific pornographic content between men and women

Pornography content	Men	Women	χ^2^
Vaginal penetration	97.00	95.70	.46
Anal penetration	76.90	38.70	74.08***
Oral sex	94.50	74.90	32.86***
Fetish/BDSM	56.80	41.30	12.21***
Violent sex/rape scenario	42.20	27.90	11.71***
Teens	69.30	24.80	104.32***
Group sex	69.80	49.30	21.87***

***p < .001

Our second hypothesis posed men would perpetrate more sexual violence than women. Prevalence results of most severe category of perpetration for the full sample are displayed in Table 3. Most people reported no perpetration (51.5% of men and 67.5% of women). Comparing the absolute prevalence per category with the prevalence of the most severe category, it appeared that participants who committed the most severe category were also responsible for the majority of perpetration in lower categories; only a limited number of men and women fell into a most severe category in the middle of the spectrum. For this reason, we dichotomized the sexual perpetration variable (Yes/No) for subsequent analyses. The questionnaire also includes the non-behavioral question “Do you think you may have ever raped someone?”. Of the men who met the behavioral criteria for rape, 63.63% denied rape when asked outright, and 87.10% of women who met the behavioral criteria for rape denied rape when asked outright**.**

**Table 3:** Absolute frequencies and mutually exclusive most severe prevalence of sexual perpetration for men and women in full samples and pornography-viewing only samples

Perpetration	Absolute frequencies				Mutually exclusive most severe			
	Men		Women		Men		Women	
	Viewing	Full	Viewing	Full	Viewing	Full	Viewing	Full
Non-perpetrator	50.80	51.50	67.00	67.50	50.80	51.50	67.00	67.50
Sexual contact	40.70	40.10	12.50	11.40	3.00	3.00	4.00	3.90
Attempted coercion	34.70	34.20	7.70	7.60	0.50	.50	.30	.20
Coercion	34.70	34.20	8.00	7.70	0.00	.00	.00	.00
Attempted rape	30.20	29.70	6.30	6.30	.50	.50	15.10	.00
Rape	33.20	32.70	8.30	7.10	33.20	32.70	8.30	7.10
Rape—non-behavioral	14.10	13.90	7.10	6.70				
Discrepancy	63.63*	63.63*	86.20*	87.10*				

*of proportion who met behavioral criteria of rape

For men, 30.7% indicated they had perpetrated multiple acts of sexual aggression. A majority (18.3%) committed sexual aggression against women only, 8.9% against men only, and 2.5% against both men and women. For women, 6.7% reported multiple instances of sexual perpetration, mostly against men only (4.4%), some against women only (1.8%) and few against both men and women (0.5%). In terms of sexual victimization, 38.1% of men indicated they had had an unwanted sexual experience, compared with 52.8% of women.

### Bivariate Associations

Correlations between variables are displayed in Table 4. For women, both frequency variables were positively associated with both peer RMA and sexual perpetration. For men, only average hours a week was linked with peer RMA. For men and women, perceiving pornography as realistic was linked with peer RMA and sexual perpetration. In terms of content, viewing vaginal penetration was negatively linked with both peer RMA and sexual perpetration in men. Oral penetration showed no association with any variables. All other content was positively and significantly associated with peer RMA and sexual perpetration for both genders, except for anal penetration, which was unrelated to sexual perpetration for men.

**Table 4:** Correlations between pornography variables and peer rape myth acceptance and sexual perpetration for men and women

	Total peer rape myth acceptance		Sexual perpetration	
Pornography	Men	Women	Men	Women
Realistic	.58***	.47***	.53***	.35***
Past six months	-.03	.18***	< .01	.13*
Hours a week	.15*	.24***	.13	.25***
Vaginal penetration	-.26***	-.09	-.17*	-.03
Anal penetration	.15*	.27***	.13	.25***
Oral sex	-.11	< -.01	-.04	.06
Fetish/BDSM	.27***	.25***	.36***	.21***
Violent sex/rape scenario	.57***	.40***	.59***	.35***
Teens	.30***	.41***	.30***	.41***
Group sex	.21**	.19***	.35***	.26***

*p < .05, **p < .01, ***p < .001

### Moderation Analyses

**Fig. 1:**
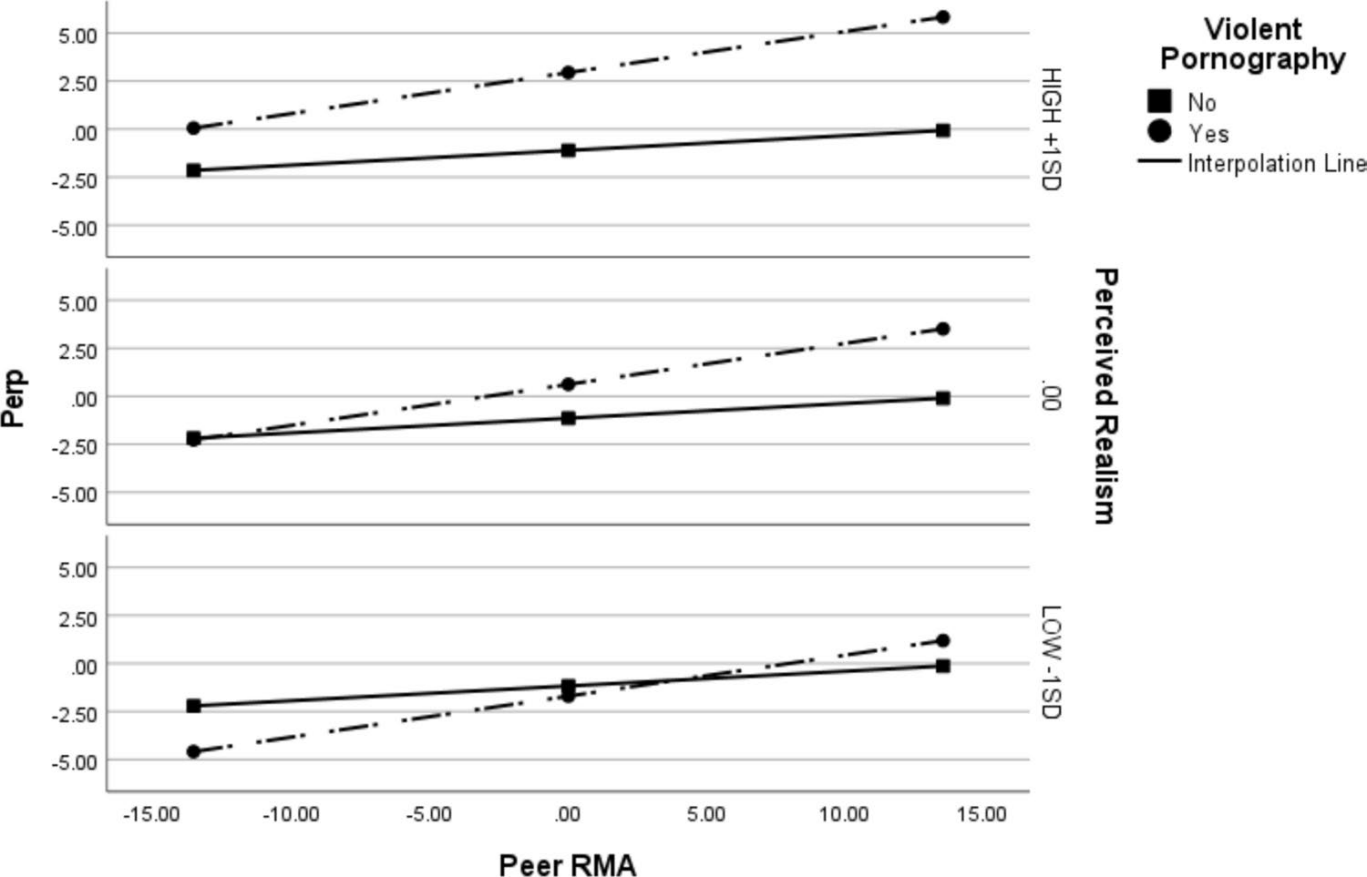
Conditional effects of peer rape myth acceptance and perceived realism on the link between viewing violent pornography and sexual perpetration for full sample of men

Finally, to assess whether viewing violent pornography would increase the risk of sexual aggression perpetration (H4), particularly if peer RMA (H5) and perceived pornography realism (H6) were high, we conducted a series of logistic parallel moderation analyses with perpetration (DV), violent pornography (IV), perceived realism (M1) and peer RMA (M2). We conducted one for the full sample of men, one for the full sample of women, and one for the sample of women who watch pornography. Results are displayed in Table 5. For men, the model was significant (Nagelkerke* R*^*2*^ = 0.71, χ^2^(5) = 111.29, *p* < 0.001). Both viewing violent pornography and peer RMA were independently associated with increased sexual perpetration. Perceived pornography realism moderated the link between viewing violent pornography and sexual perpetration, such that this link was stronger at higher levels of perceived realism. Specifically, at low RMA (-1SD) violent pornography viewing was not significantly associated with sexual perpetration, regardless of level of perceived realism. At mean peer RMA, the relationship between violent pornography viewing and sexual perpetration was significant only when perceived realism was at mean (Effect_mean_realism_ = 1.76, *p* = 0.007) or high (+ 1SD; Effect_high_realism_ = 4.05, *p* < 0.001) levels. The same pattern was observed at high peer RMA (+ 1SD): the relationship between violent pornography viewing and sexual perpetration was significant at both mean (Effect_mean_realism_ = 3.61, *p* = 0.006) and high (+ 1SD; Effect_high_realism_ = 5.90, *p* < 0.001) levels of perceived realism. These results are displayed in Fig. 1.

**Table 5:** Logistic parallel moderation analyses for the moderating effect of perceived realism and peer rape myth acceptance on the link between violent pornography viewing and sexual perpetration for men, women, and pornography-viewing women only

	Men				Women full sample				Women pornography-viewing only			
	B	beta	Wald χ^2^	Odds ratio	B	beta	Wald χ^2^	Odds ratio	B	beta	Wald χ^2^	Odds ratio
Violent pornography	1.76***	.79*	7.35	5.82	.02	−.01	< .01	1.02	.11	−.03	.02	1.12
Realism	.03	.98*	.01	1.03	.40*	.53**	8.24	1.50	.23	.40*	1.01	1.25
Peer RMA	.08*	1.80***	6.28	1.08	< .01	.39*	.08	1.01	.01	.44*	.31	1.01
Violent pornography x realism	.14**	1.13***	7.00	1.15	.68	.28	11.61	1.97	.85	.34	5.25	2.35
Violent pornography x Peer RMA	1.85	.91	6.28	6.36	.14**	.58**	1.63	1.15	.13**	.55**	10.36	1.14

*B* = unstandardized beta coefficient, beta = standardized beta coefficient, Wald χ^2^ = Wald chi-square; although the unstandardized coefficients show the actual change in the dependent variable (in original units), the standardized betas offer a clearer view of the relative importance of each predictor, especially when different scales are involved, which explains the discrepancy in significance between B and beta. However, the standardized coefficients for dichotomous variables might not be as informative, as standardization (which involves scaling by the standard deviation) typically applies to continuous variables. *p < .05, **p < .01, ***p < .001

**Fig. 2:**
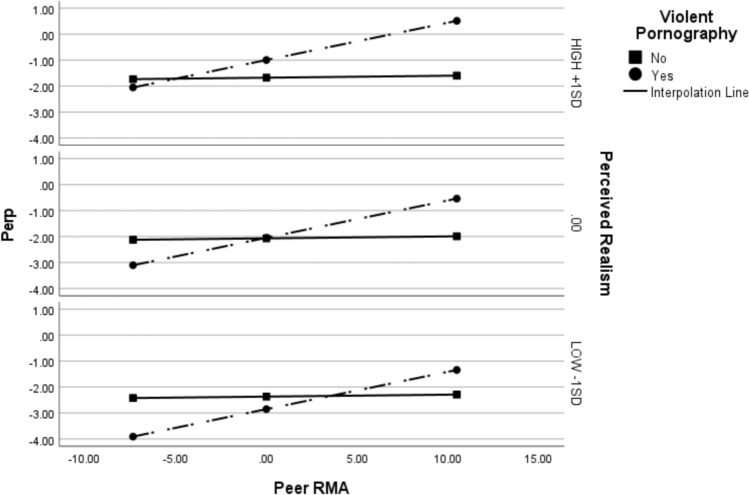
Conditional effects of peer rape myth acceptance and perceived realism on the link between viewing violent pornography and sexual perpetration for full sample of women

We then ran the same analysis on the full sample of women. The model was significant (Nagelkerke* R*^*2*^ = 0.36, χ^2^(5) = 226.89, *p* < 0.001). Perceived pornography realism was independently associated with increased sexual perpetration, and there was a significant interaction between viewing violent pornography and peer RMA. Specifically, at high levels of peer RMA (+ 1SD), viewing violent pornography was significantly associated with sexual perpetration when perceived realism was at mean (Effect_mean_realism_ = 1.45, *p* = 0.01) or high (+ 1SD; Effect_high_realism_ = 2.11, *p* < 0.001). These results are displayed in Fig. 2.

**Fig. 3:**
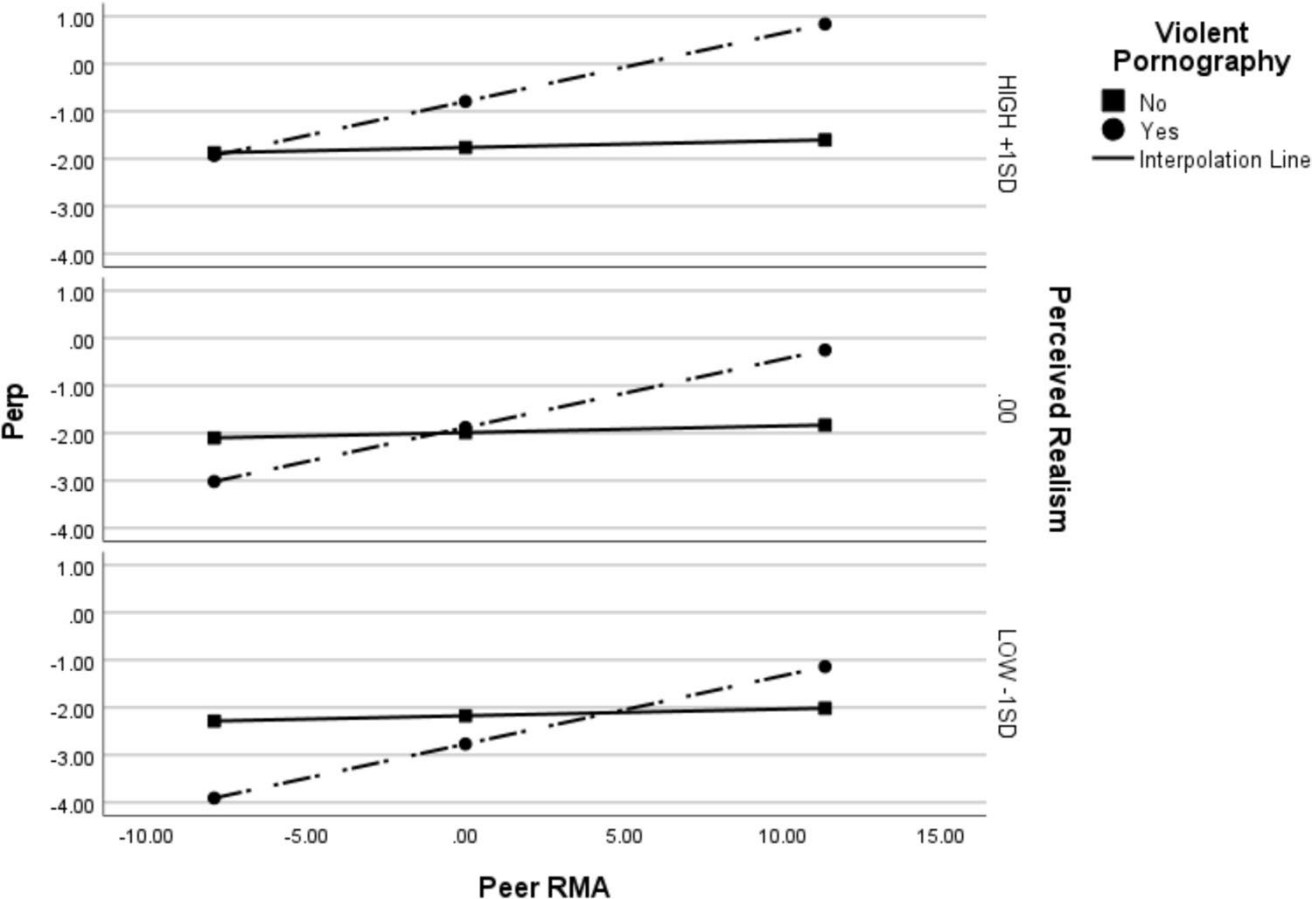
Conditional effects of peer rape myth acceptance and perceived realism on the link between viewing violent pornography and sexual perpetration for women who report viewing pornography

Finally, we ran the same analysis on the sample of women, excluding those who did not view pornography. Again, the model was significant (Nagelkerke* R*^*2*^ = 0.39, χ^2^(5) = 189.68, *p* < 0.001). Only the interaction between peer RMA and violent pornography was significant, indicating that the relationship between violent pornography and sexual perpetration depends on peer RMA levels. At high peer RMA (+ 1SD), viewing violent pornography was significantly associated with increased sexual perpetration, but only if perceived realism was mean (Effect_mean_realism_ = 1.58, *p* = 0.01) or high (+ 1SD; Effect_high_realism_ = 2.44, *p* < 0.001) levels. These results are displayed in Fig. 3.

## Discussion

The aim of this study was to examine the moderating effects of peer rape myth acceptance (H5) and perceived pornography realism (H6) on the link between consumption of violent pornography and self-reported sexual perpetration in men and women (H4). Consistent with previous research, pornography use seems normalized for both men and women. However, gender differences in attitudes toward pornography and diversity of content reflect cultural scripts that dictate how men and women engage with explicit sexual media. The greater perceived realism reported by our male participants may stem from a greater alignment of pornographic content with traditional masculine scripts about sexual interactions (Bareket & Shnabel, 2020). On the other hand, a discrepancy between women’s real-life experiences and pornography may explain their lower perceived realism (Peter & Valkenburg, 2016). This gender difference is consistent with research suggesting that media (including pornography) may reinforce distorted gender norms, and men are more likely to adopt these norms as realistic (Ward, 2002).

The men in our sample perceived their peers to disagree with rape myths, compared to women who indicated their peers would strongly disagree. While this is a small difference, even subtle differences can be impactful. Much research in recent years has pointed out that for rape culture to be challenged, it needs to be challenged in male spaces (de Roos & Jones, 2022). Within male social groups, rape myths may be more subtly tolerated or less openly challenged, which is often tied in with a fear of being ridiculed (Berkowitz et al., 2022). Indeed, all-male social circles often include dynamics where distorted gender norms and rape-permissive attitudes are implicitly reinforced through jokes or subtle approval of problematic behavior (Banyard et al., 2014; Bohner et al., 2006). Bohner et al. (2006) noted that in the absence of vocal rejection of rape myths, group norms in male-dominated groups could be misperceived. However, this study did not ask participants to specify the gender of their three closest friends, which should be included in future research to determine to what extent people’s perception of their peers’ attitudes is consistent with the general trend that men are more accepting of rape myths than women. Similarly, participants should be asked about their own RMA, to examine the presence of “pluralistic ignorance”; the false belief that peers share similar beliefs when they do not (Berkowitz et al., 2022).

Regarding perpetration, our results indicated that placing individuals in mutually exclusive categories of their most severe behavior created a dichotomous variable of non-perpetration and perpetration. This was because if someone reported both a less severe act (e.g., fondling) and a more severe act (e.g., rape), they were categorized according to the most severe act. The individuals who reported rape were often the same ones responsible for the prevalence of the less severe categories of perpetration. This overlap between severe and less severe acts suggests some participants exhibit a behavioral continuum. Such a continuum is consistent with behavioral progression, for example, Lisak and Miller (2002) described a subset of perpetrators who engaged in a range of sexually aggressive behaviors that escalated in severity over time. Similarly, a recent study by the UK Ministry of Justice (2023) identified patterns of escalation in severity over time, highlighting the need for early detection and intervention. Future research should incorporate longitudinal designs that can track potentially escalating behavior over time.

Interestingly, we found discrepancies in participants’ answers to behavioral questions that would meet the definition of rape, and to the explicit question: “Do you think you may have ever raped someone?”. Women who met the behavioral criteria of rape were unlikely to admit they may have raped someone when asked outright, which suggests the myth that women cannot rape may prevail. This myth is reflected in legal definitions of rape which vary across jurisdictions and may not incorporate female rape committed against men (Fisher & Pina, 2013). In the context of sexual victimization at a university, female-perpetrated sexual violence remains highly stigmatized, with impacts victims’ ability and willingness to seek help (Gambardella et al., 2020).

As hypothesized, perceived realism of pornography was significantly associated with both peer RMA as well as sexual perpetration, for men and women. In terms of pornographic content, vaginal penetration had a negative relation with both peer RMA and sexual perpetration for men. For both genders, oral sex had no association to either variable. The association for anal penetration was inconsistent. This suggests more “mainstream” pornography is unlikely to be linked with sexual perpetration or peer RMA, which is consistent with prior research (Burnay et al., 2022). Conversely, more deviant content including violent sex and rape scenarios were associated with peer RMA and sexual perpetration for men and women. These findings correspond to those of several meta-analyses that found deviant sexual interest is a strong predictor of sexual offending behavior (Seto & Lalumiere, 2010) and sexual recidivism (Hanson & Morton-Bourgon, 2005; McCann & Lussier, 2008).

For men, viewing violent pornography, peer RMA and perceived realism had positive, significant main effects on sexual perpetration. For the full sample of women and the pornography—viewing only sample of women—we found main effects of perceived realism and peer RMA but not of violent pornography. This finding suggests men may be particularly susceptible to internalizing harmful stereotypes portrayed in pornography, which increases the risk of them engaging in sexual violence. This is likely due to stereotypical content that portrays traditional sexual scripts with men as dominant aggressors, and women as passive submissives (Klein et al., 2019). Further, when it comes to risky sexual behavior, adolescent boys and men are more susceptible to influence from their peers, and they are more likely to be motivated by status than connection within their peer groups compared with women (Rose & Rudolph, 2006).

Interestingly, when we looked at the moderator effects, for men, we found the link between violent pornography and sexual violence perpetration was affected by perceived realism. This finding underlines the importance of perceived realism in dictating the extent to which pornographic content is internalized and normalized. This moderating effect of perceived realism is consistent with prior research showing sexual violence perpetration in men may in part be explained by how realistic they perceive the media they consume to be (e.g., Malamuth et al., 2021; Peter & Valkenburg, 2016). For both samples of women, we found the moderating effect of peer RMA was significant. This finding indicates that for women, their social environment is a more important influence on their sexual behavior. The gender difference highlights a different mechanism by which information becomes part of sexual scripts. Our findings partially align with those of Krahé et al. (2021). In their study, they found pornography realism positively predicted risky sexual behavior and acceptance of sexual coercion, which in turn was linked with sexual aggression perpetration. However, they did not find any gender differences. These discrepancies may be due to their use of pornography in general as a factor, whereas we focused exclusively on violent pornography.

### Practical Implications

Our findings highlight the potential for violent pornography to substantially increase risk of sexual violence perpetration among male students in particular. They further underline the interaction with both perceived realism for men and peer RMA for women. These findings translate into practical implications for prevention and intervention on university campuses but also much earlier, for example in secondary school. First, prevention should contain media literacy education to challenge perceived realism. In a longitudinal study, Vandenbosch and van Oosten (2017) found that porn literacy education weakened the link between sexually explicit Internet material and sexist views in teenagers and emerging adults. They posed such education should be part of schools’ sex education programs to counterbalance the potentially harmful effects of pornographic content on sexual development. Importantly, such education should take a non-judgmental perspective toward pornography and instead focus on the development of critical thinking skills surrounding the portrayal of sex and gender dynamics in pornography (Dawson et al., 2020) and challenging the perceived realism of such content. Wright et al. (2022) found that the range of pornography exposure did not affect respondents’ emulation of common sexual scripts portrayed in such content, i.e., condomless sex, when perceived pornography realism was low. However, there was an effect when perceived realism was high. These findings align with the effectiveness of a web-based sexual health program focused on critiquing unrealistic media messages on sexual behavior, which led to a reduction in risky sexual behaviors among college students (Scull et al., 2018).Whereas most such interventions focus on pornography in general, it would be worthwhile paying specific attention to explicitly violent pornography, and diluting any negative effects through a more nuanced, gender-balanced discussion of pornography.

Secondly, the influence of peer groups should be a focus of attention. Within a university context, a first step would be to stimulate and facilitate the mixing of genders, particularly in traditionally male-dominated programs of study. More broadly, extracurricular activities with an explicit gender-balance may alleviate some of the negative effects of peer influence in this area. Building on this, a challenge to commonly held, harmful attitudes toward sexual violence should begin from within. In any group, it falls to in-group members to dictate what is (un)accepted behavior (Pettigrew, 1961; Turner, 1991). As such, the most effective way to reshape social norms surrounding sexual violence is through men calling out men for inappropriate behavior (Fabiano et al., 2003). Stein (2007) found that college men believed that their close friends held more rape-supportive attitudes and behaviors than they themselves did. Nevertheless, perceived peer beliefs about rape still influenced respondents’ willingness to prevent rape. These findings underscore the influence of social norms, wherein even when incorrectly perceived, they can shape individuals’ attitudes and behaviors (Berkowitz et al., 2022). Strategies to prevent sexual violence should incorporate interactive discussions that allow men to become aware of the true norms held by their peers and to potentially positively change their own attitudes and behaviors. Indeed, a study by Bohner et al. (2006) reported that when college men with high self-reported RMA were told that their peers rejected rape myths, their reported proclivity to perpetrate sexual violence was lower than when told their peers endorsed rape myths (Bohner et al., 2006). Through redefining constructs like masculinity to incorporate prosocial attitudes and reject derogatory views from male identity, a shift may take place (Gottell & Dutton, 2016; Harlow et al., 2021). Such interventions should also include women, as they are clearly also susceptible to peer influences that promote harmful ideas about sexual violence.

### Strengths and Limitations

Strengths of this study include asking participants about a broad range of sexual violence perpetration and pornography genres. In terms of limitations, firstly, sexual perpetration in this sample was dichotomous, which made it impossible to take severity of sexual violence perpetration into account. Future research may use a larger sample and focus more explicitly on “milder” forms of sexual violence perpetration to determine how violent pornography and peer RMA impact the lower end of the spectrum. Secondly, this study focused on “traditional,” audiovisual pornography. Particularly women may be more likely to engage with different forms of pornography such as erotic literature. Importantly, erotic literature also tends to adhere to traditional sexual scripts (Menard & Cabrera, 2011), and thus should be included when studying the effects of sexually explicit material. Future studies may wish to employ a broader definition of pornography to include more diverse media. Thirdly, directionality should be interpreted with caution. It is likely that people with preexisting interest in violent sex seek out pornographic content that aligns with this interest. This limits our ability to conclude that violent pornography contributes to an increased likelihood of sexual violence perpetration. Fourthly, perceived realism may be particularly relevant for sexual behaviors in more sensitive developmental periods, before people have “real-life” sexual experiences (Wright, 2011; Wright & Stulhofer, 2019). Given our participants were young adults, perceived pornography realism may be less relevant for this age group. Finally, with regard to peer RMA, we asked participants to think of their three closest friends when filling out this questionnaire. We did not ask them about the gender of these friends, and thus we are unable to conclude that men mostly associate with men, which would drive the effect of peer RMA as men tend to score higher on RMA. Given the findings relating to isolated circles of male friends and how such an environment increases the risk of sexual violence perpetration and positive attitudes toward sexual violence, future research should take this into consideration. Further, participants reported their perceptions of their peers, which may not be accurate. However, we would argue that in this context, the accuracy of such perceptions is less relevant than how the individual perceives their peers to view issues related to sexual violence. Finally, the practical gender difference in peer RMA was small, which suggests future studies might use an updated version of the IRMA (i.e., McMahon & Farmer, 2011) to capture more subtle rape myths.

### Conclusion

In a large study of university students, we found that viewing violent pornography increased the risk of men’s self-reported sexual violence perpetration. This effect was exacerbated if men perceived pornography to be realistic. For women, viewing violent pornography was not independently linked with sexual violence perpetration, but this link emerged if they perceived their peers to be more accepting of rape myths. These findings highlight the importance of addressing risk factors such as violent pornography consumption and strengthening protective factors such as media literacy and the challenging of rape myths, particularly in peer groups. Tailoring interventions to address the gendered patterns in sexual script formation could be a more effective approach to preventing sexual violence.

## Data Availability

Available upon request.
